# A Lever Coupling Mechanism in Dual-Mass Micro-Gyroscopes for Improving the Shock Resistance along the Driving Direction

**DOI:** 10.3390/s17050995

**Published:** 2017-04-30

**Authors:** Yang Gao, Hongsheng Li, Libin Huang, Hui Sun

**Affiliations:** 1 School of Instrument Science and Engineering, Southeast University, Nanjing 210096, China; 230139244@seu.edu.cn (Y.G.); huanglibin@seu.edu.cn (L.H.); 220142642@seu.edu.cn (H.S.); 2 Key Laboratory of Micro-Inertial Instruments and Advanced Navigation Technology, Ministry of Education, Nanjing 210096, China

**Keywords:** coupling mechanism, MEMS, shock resistance, mechanical design, silicon micro-gyroscope

## Abstract

This paper presents the design and application of a lever coupling mechanism to improve the shock resistance of a dual-mass silicon micro-gyroscope with drive mode coupled along the driving direction without sacrificing the mechanical sensitivity. Firstly, the mechanical sensitivity and the shock response of the micro-gyroscope are theoretically analyzed. In the mechanical design, a novel lever coupling mechanism is proposed to change the modal order and to improve the frequency separation. The micro-gyroscope with the lever coupling mechanism optimizes the drive mode order, increasing the in-phase mode frequency to be much larger than the anti-phase one. Shock analysis results show that the micro-gyroscope structure with the designed lever coupling mechanism can notably reduce the magnitudes of the shock response and cut down the stress produced in the shock process compared with the traditional elastic coupled one. Simulations reveal that the shock resistance along the drive direction is greatly increased. Consequently, the lever coupling mechanism can change the gyroscope’s modal order and improve the frequency separation by structurally offering a higher stiffness difference ratio. The shock resistance along the driving direction is tremendously enhanced without loss of the mechanical sensitivity.

## 1. Introduction

With the development of MEMS (microelectromechanical systems) technology, the performances of MEMS devices are greatly improved, and now, the sensors are widely used in various fields. For many applications, such as in military and aerospace applications, the sensors not only have to perform with a high degree of stability, but also have to withstand a higher level of shock. Therefore, it is crucial to improve the reliability and survivability of MEMS devices in a shock environment [1,2].

Micro-gyroscopes, as one kind of MEMS device, are silicon-based sensors, which can detect input angular rate information through the Coriolis acceleration. In comparison with their conventional counterparts, micro-gyroscopes have many advantages, such as small size, low cost, low power consumption, micro-volume, tiny weight and batch fabrication. With the advancement of the architecture, measuring and controlling technology, micro-gyroscopes have achieved a rapid development in the past several decades. At present, they are widely used in many fields, including the automotive industry, consumer electronics, robot control systems, aerospace navigation and military weapons [3,4].

In high shock environments, the impact loads could cause the large nonlinear displacement of the elastic structures, which will result in the nonlinear stress strain behavior of the material and will lead to collisions between the movable structure and the fixed structure. In addition, the massive displacement will produce heavy stress or strain in the structure, especially in its weak structures (mostly elastic beams) [5,6]. The cracking, chipping and fracture problems caused by the collision and excessive deformation are the common failure modes of the structure. Unlike failure in large-scale devices, the other important sources of failure in MEMS are stiction and electric short circuits due to the contact between the movable part and the stationary part or the substrate [7]. The shock response and failures of micro-structures have been studied by many researchers. Fang et al. investigated the half-sine shock response of a micro-cantilever using a beam model. The displacement and bending stresses of the micro-beam were calculated by the assumed modes method [8]. Li et al. used the equivalent lumped spring-mass model to approximate the dynamic response of micro-structures to study the motion of MEMS accelerometers during the drop tests. Both SDOFand the distributed-parameter model were used to calculate maximum deflection of the cantilever and hinged-hinged beam [9]. In [10], the authors experimentally characterized the shock response of commercial accelerometers. The authors in [11] provided details of the dynamic response under severe multi-axial single-pulse shock load, which was performed using the finite element tool ABAQUS with nonlinear dynamics procedures. In [12,13], the authors proposed a multi-scale numerical approach to compute the stress state induced in polysilicon MEMS sensors by accidental drops and to detect eventual failure locations. Through Monte Carlo simulations, some effects of polysilicon micro-structure on the failure mode are elucidated. They presented a two-scale approach to model drop-induced failures of polysilicon MEMS sensors in [14], which addressed the whole tracking of the failure mechanism.

So far, researchers have attempted to improve the reliability of the MEMS devices in shock environments and have carried out many achievements. Enhancing the robustness of the weak structures in MEMS devices, by the optimization of the beams and structure design for stress relief, is one common method. A circular arc step structure at the corner of a notch structure was given in [15]. The maximal stress of the structure was significantly reduced by this design, and the high shock resistance ability of the MEMS gyroscope exceeded 10,000 g. In [16], the authors reported the spring corner designs to improve the reliability for a MEMS actuator. The experimental results show that the MEMS device designed by these principles can survive a 500 g (gravity acceleration) shock test. However, this method improves the anti-shock performance slightly [5]. Another widely-used method that enhances the anti-shock performance is the use of stoppers, including solid stoppers and elastic stoppers. This approach is widely employed in MEMS accelerometers. The authors in [17,18] presented two shock protection solutions of different elastic stoppers, nonlinear-spring shock stoppers and soft-coating shock stoppers, and compared the shock protection performance of the elastic stoppers with that of the hard stoppers by experiments. Simulation and experimental results clearly demonstrated that elastic stoppers offer superior shock protection compared to conventional hard ones. A mixed shock protection method by using flexible decoupling and the stopper frame for Colibrys sandwich accelerometers was given in [19]. In [20], the authors showed two-level elastic stoppers, which had a significant effect on improving the anti-shock performance. A kind of double-cascaded stopper to mitigate high-frequency shock failure was presented in [6]. Shock tests showed that accelerometers with flexible stoppers could resist more than 10,000 g shock with about a 100-μs pulse width, while the double-cascaded stopper was more robust to high-frequency shocks. However, these stoppers may only provide marginal protection because they themselves may produce secondary impact sources (such as subsequent shocks) that may cause fracture, debris and performance shifts in the device [18].

The effective shock protection must minimize the impulse response displacement, as well as minimize the impulse forces delivered to the microstructure. For a dual-mass silicon micro-gyroscope, a useful method is to raise the in-phase mode frequency, which can further improve its shock resistance. In [20], the authors not only equipped their gyroscopes with two level elastic stoppers, but also raised the in-phase mode frequency. Shock experiments indicated that the shock resistance of the gyroscope along the *X*-axis had been increased from 2000 g to 15,000 g. However, generally, for a dual-mass silicon micro-gyroscope coupled by elastic beams, the in-phase mode frequency is lower than and close to the anti-phase mode frequency, which is the operational mode frequency. The improving of in-phase mode frequency will also raise the anti-phase mode frequency, which will influence the mechanical sensitivity. Therefore, changing the modal order by switching the in-phase mode frequency above the anti-phase one and improving the frequency separation are important to improve the shock resistance without sacrificing any sensitivity. Hence, it is vital to designing and analyzing the coupling mechanism. In [21], an anchored coupling ring spring linked by four linear beams was presented. The simulation showed that the in-phase mode frequency of the anchored coupling silicon micro-gyroscope was increased by 48.7%, while the anti-phase mode frequency was improved a little. The vibration outputs were reduced by 74.8% and 88.1% in the anti-phase mode and in-phase mode, respectively. A new micromachined tuning fork gyroscope with an anchored diamond coupling mechanism was proposed in their recent paper, which increased the in-phase mode frequency to be 108.3% higher than the anti-phase one [22]. A self-rotation ring structure for connecting two sensing proof masses was designed and simulated in [23], and the in-phase sensing mode was suppressed by using it.

The ring and diamond coupling structures presented in the published materials could change the modal order and improve the frequency separation, but these structures have very stringent machining precision requirements. Moreover, the shock resistance has not been discussed in these articles. This paper presents a lever coupling mechanism, which can not only improve the gyroscope’s shock resistance along the driving direction, but also will not sacrifice any mechanical sensitivity. Compared with the ring and diamond structures, this structure is simpler and is easier to process. Section 2 discusses the relationship between the mechanical sensitivity and the drive mode frequency. Section 3 describes the shock response of the ideal dual-mass silicon micro-gyroscope. The relationship between the shock response and in-phase frequency in driving direction is analyzed. Section 4 presents the design and analysis of the lever coupling mechanism. In addition, a silicon micro-gyroscope with this proposed lever coupling mechanism based on our previous structure is described. In Section 5, the modal order, temperature performance and the shock resistance are analyzed by the FEM method. Section 6 discusses the whole paper.

## 2. Analysis of the Mechanical Sensitivity

Neglecting the nonlinearity, which is derived from the large deformation of the beams, the dynamic equations of a dual-mass silicon micro-gyroscope can be expressed as:

(1) $$m_{x}\ddot{x}+c_{x}\dot{x}+k_{x}x=F_{d},$$

(2) $$m_{y}\ddot{y}+c_{y}\dot{y}+k_{y}y=F_{c},$$

where *x*, *y* are the mass displacements in the drive and sense direction; $m_{x}$, $m_{y}$ represent the mass along the *X*-axis and *Y*-axis; $c_{x}$, $c_{y}$ represent the damping coefficients along the *X*-axis and *Y*-axis; $k_{x}$, $k_{y}$ represent the stiffness along the *X*-axis and *Y*-axis; $F_{d}$, $F_{c}$ are the driving force and the Coriolis force, respectively, and can be expressed by:

(3) $$F_{d}=A_{f}sin\omega t,F_{c}=2m_{y}\Omega \dot{x},$$

where $A_{f}$ is the amplitude of the driving force; ω is the angular frequency of the driving force; Ω is the input angular velocity.

The solutions of (1) and (3) could be obtained as:

(4) $$x\left(t\right)=\frac{A_{f}/m_{x}}{\omega_{d}^{2}\sqrt{{\left(1-\frac{\omega^{2}}{\omega_{d}^{2}}\right)}^{2}+{\left(\frac{1}{Q_{d}^{2}}{\left(\frac{\omega }{\omega_{d}}\right)}^{2}\right)}^{2}}}sin\left(\omega t-\varphi_{d}\right),$$

where $\varphi_{d}=tan^{-1}\frac{2\zeta_{d}\omega_{d}\omega }{\omega_{d}^{2}-\omega^{2}}$; $\omega_{d}$ is the drive mode frequency, which is the operational frequency; $Q_{d}$, $\zeta_{d}$ are the quality factor and the damping ratio of the drive mode, respectively. The solutions of (2)–(4) could be obtained as:

(5) $$\begin{array}{cc} y(t) & =A_{y}cos\left(\omega t-\varphi_{d}-\varphi_{s}\right)\\ & =\frac{2m_{c}\Omega \omega A_{F}}{m_{x}m_{y}\sqrt{{\left(\omega^{2}-\omega_{d}^{2}\right)}^{2}+\frac{\omega^{2}\omega_{d}^{2}}{Q_{d}^{2}}}\sqrt{{\left(\omega^{2}-\omega_{s}^{2}\right)}^{2}+\frac{\omega^{2}\omega_{s}^{2}}{Q_{s}^{2}}}}cos\left(\omega t-\varphi_{d}-\varphi_{s}\right), \end{array}$$

where $\varphi_{s}=tan^{-1}\frac{2\zeta_{s}\omega_{s}\omega }{\omega_{s}^{2}-\omega^{2}}$; $\omega_{s}$ is the sense mode frequency; $Q_{s}$, $\zeta_{s}$ are the quality factor and the damping ratio of the sense mode, respectively.

The mechanical sensitivity can be obtained as follows:

(6) $$\begin{array}{cc} S_{y} & =\frac{A_{y}}{\Omega }\\ & =\frac{2m_{c}\omega A_{F}}{m_{x}m_{y}}\cdot \frac{1}{\sqrt{{\left(\omega^{2}-\omega_{d}^{2}\right)}^{2}+\frac{\omega^{2}\omega_{d}^{2}}{Q_{d}^{2}}}}\cdot \frac{1}{\sqrt{{\left(\omega^{2}-\omega_{s}^{2}\right)}^{2}+\frac{\omega^{2}\omega_{s}^{2}}{Q_{s}^{2}}}}. \end{array}$$

Ideally, $\omega =\omega_{d}$, Δω is defined as the frequency difference between the drive mode and the sense mode, which is given by $\Delta \omega =\omega_{d}-\omega_{s}$. (6) can be expressed as:

(7) $$\begin{array}{cc} S_{y} & =\frac{2m_{c}\omega_{d}A_{F}}{m_{x}m_{y}}\cdot \frac{Q_{d}}{\omega_{d}^{2}}\cdot \frac{1}{\sqrt{{\left(2\omega_{d}\Delta \omega -{\left(\Delta \omega \right)}^{2}\right)}^{2}+\frac{\omega_{d}^{2}{\left(\omega_{d}-\Delta \omega \right)}^{2}}{Q_{s}^{2}}}}\\ & =\frac{m_{c}f_{d}A_{F}}{8\pi^{3}m_{x}m_{y}}\cdot \frac{Q_{d}}{f_{d}^{2}}\cdot \frac{1}{\sqrt{{\left(2f_{d}\Delta f-{\left(\Delta f\right)}^{2}\right)}^{2}+\frac{f_{d}^{2}{\left(f_{d}-\Delta f\right)}^{2}}{Q_{s}^{2}}}}, \end{array}$$

where $f_{d}=\frac{1}{2\pi }\omega_{d}$; $\Delta f=\frac{1}{2\pi }\Delta \omega$.

**Figure 1:**
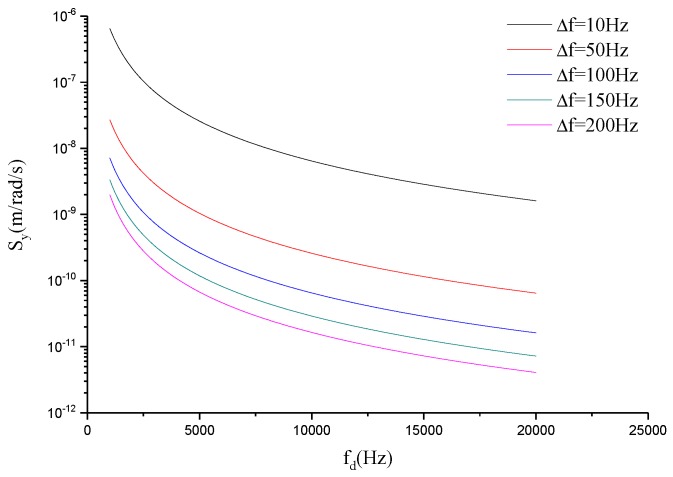
The curves of the mechanical sensitivity related to Δf and $f_{d}$.

$m_{c}$, $m_{x}$ and $m_{y}$ are fixed when the structure form is assured. $Q_{d}$ and $Q_{s}$ are mainly decided by processing and packaging processes. Then, the mechanical sensitivity is primarily determined by the values of $f_{d}$ and Δf. Figure 1 illustrates the relationship between the mechanical sensitivity, $f_{d}$, and Δf.

It can be seen that the mechanical sensitivity is increased with the reduction of |Δf| and is decreased with the rising of the drive mode frequency. However, because the working bandwidth of the gyroscope is estimated as BW=0.54|Δf|, the value of |Δf| cannot be too small for the requirement of the working bandwidth. Therefore, in order to get a higher mechanical sensitivity, $f_{d}$ should be as low as possible.

## 3. Shock Load and Shock Response Analysis

### 3.1. Shock Load

**Figure 2:**
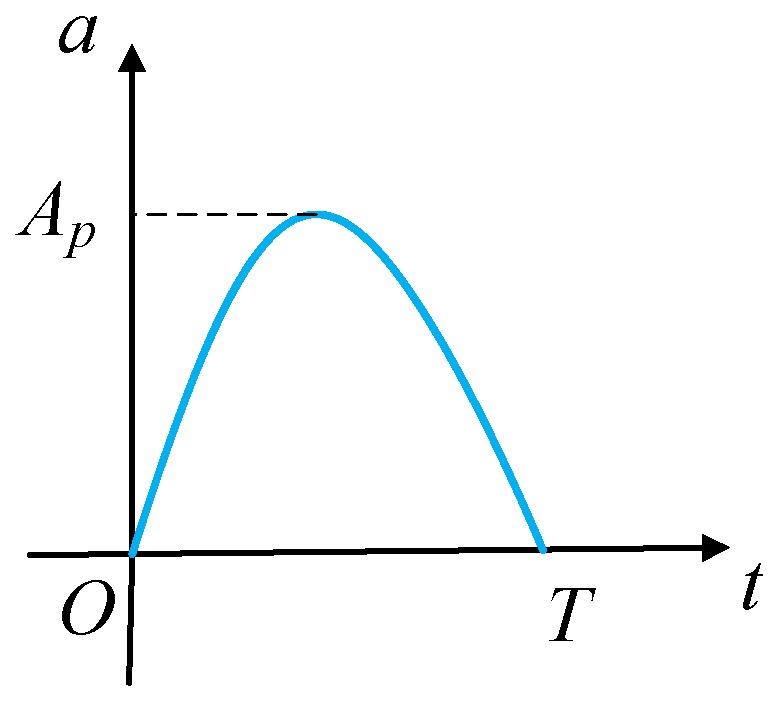
Shock load.

The shocks in shock environments are invariably irregular in pulse shape, jagged in spectral characteristics and variable from one occurrence to another. Such shock loads can be conveniently approximated by a series of simple shock pulses. A classical form of such a pulse is the half-sine waveform, as is shown in Figure 2, which can be expressed as:

(8) $$a\left(t\right)=\left\{\begin{array}{cc} A_{p}sin\omega t & 0\leq t\leq T,\\ 0 & t\geq T, \end{array}\right.$$

where ω=π/T, *T* is the duration time of the shock load and $A_{p}$ is the peak acceleration of the shock load.

### 3.2. Shock Response Analysis

**Figure 3:**
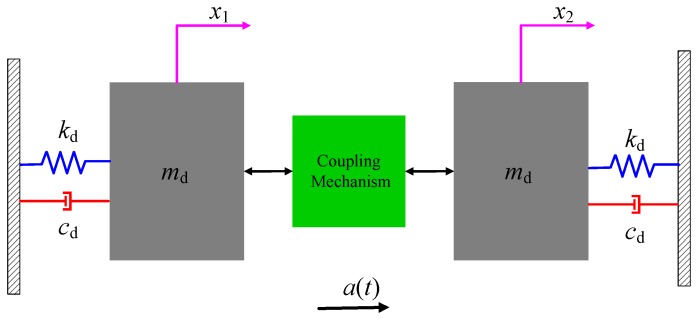
Schematic diagram of a dual-mass micro-gyroscope with drive mode coupled.

For a dual-mass micro-gyroscope, as is shown in Figure 3, the kinetic equation along the driving direction under the base acceleration load can be expressed as:

(9) $$\left[m\right]\left\{\ddot{x}\left(t\right)\right\}+\left[c\right]\left\{\dot{x}\left(t\right)\right\}+\left[k\right]\left\{x\left(t\right)\right\}=-a\left(t\right){\left\{\begin{array}{cc} m_{d} & m_{d} \end{array}\right\}}^{T},$$

where [m], [c], [k] are the second order mass matrix, damping matrix and stiffness matrix, respectively; x(t) represents the displacement vector of coriolis masses.

The stiction and the electric short circuits due to the contact between the movable part and the stationary part or the substrate are important sources of failure in MEMS, which are different from the large-scale devices. Then, the displacement of the devices should be lower than the gap between the movable part and the stationary part or the substrate. For the MEMS gyroscope, the gap is about several micrometers to several tens of micrometers, and the design of the support beam and the structural form generally ensures that the structure has a good linearity at this level of displacement. Therefore, ignoring the nonlinear effect, (9) can be simplified and solved by the modal superposition method. This method can get the analytical solution of the shock response, which is convenient for analyzing intuitively. The solving process is described in Appendix A.

The shock response, which is compatible with the analytical solution [24], is given by:

(10) $$\begin{array}{c} x\left(t\right)=\left\{\begin{array}{cc} \begin{array}{c} -\frac{A_{p}e^{-\zeta_{1}\omega_{d-in}t}}{\omega_{d-in}\sqrt{1-\zeta_{1}^{2}}}\frac{1}{\omega^{4}-2\omega^{2}\omega_{d-in}^{2}\left(1-2\zeta_{1}^{2}\right)+\omega_{d-in}^{4}}\{2\zeta_{1}\omega \omega_{d-in}^{2}\sqrt{1-\zeta_{1}^{2}}-\\ e^{\zeta_{1}\omega_{d-in}t}cos\left(\omega t\right)+\omega_{d-in}^{3}e^{\zeta_{1}\omega_{d-in}t}\left[{\sqrt{1-\zeta_{1}^{2}}}^{3}+\zeta_{1}^{2}\left(1-\zeta_{1}^{2}\right)-\sqrt{1-\zeta_{1}^{2}}\right]sin\left(\omega t\right)+\\ \left[\omega^{3}-\omega \omega_{d-in}^{2}\left(1-\zeta_{1}^{2}\right)+\omega \omega_{d-in}^{2}\zeta_{1}^{2}\right]sin\left(\omega_{d-in}\sqrt{1-\zeta_{1}^{2}}t\right)\} \end{array} & 0\leq t\leq T,\\ \begin{array}{c} -\frac{A_{p}e^{-\zeta_{1}\omega_{d-in}t}}{\omega_{d-in}\sqrt{1-\zeta_{1}^{2}}}\frac{1}{\omega^{4}-2\omega^{2}\omega_{d-in}^{2}\left(1-2\zeta_{1}^{2}\right)+\omega_{d-in}^{4}}\cdot \\ \{2\zeta_{1}\omega \omega_{d-in}^{2}\sqrt{1-\zeta_{1}^{2}}\left[cos(\omega_{d-in}\sqrt{1-\zeta_{1}^{2}}t)+e^{\zeta_{1}\omega_{d-in}t}cos\left(\omega_{d-in}\sqrt{1-\zeta_{1}^{2}}\left(t-T\right)\right)\right]+\\ \left[\omega^{3}-\omega \omega_{d-in}^{2}\left(1-\zeta_{1}^{2}\right)+\omega \omega_{d-in}^{2}\zeta_{1}^{2}\right][sin\left[\omega_{d-in}\sqrt{1-\zeta_{1}^{2}}t\right]+\\ e^{\zeta_{1}\omega_{d-in}t}sin\left(\omega_{d-in}\sqrt{1-\zeta_{1}^{2}}\left(t-T\right)\right)]\} \end{array} & t\geq T. \end{array}\right. \end{array}$$

In order to realize the relationship between the shock response and the in-phase frequency more intuitively, a numerical simulation has been done by MATLAB software. The parameters used for the simulation are shown in Table 1.

**Figure 4:**
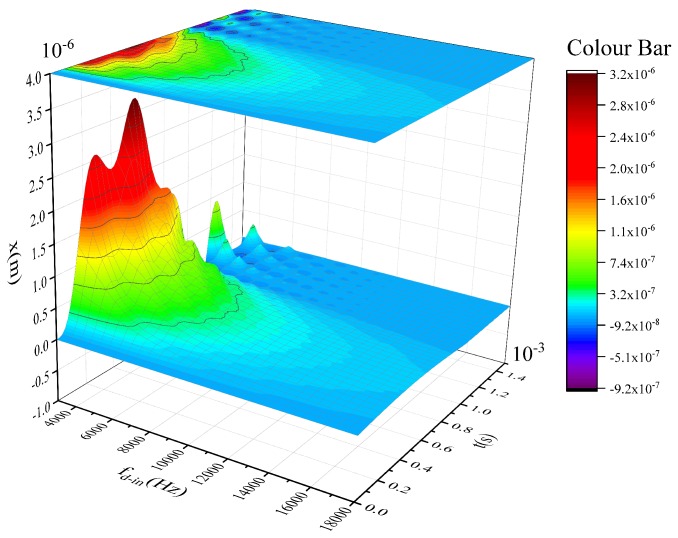
The shock response related to the in-phase frequency.

Figure 4 shows the simulation results. It can be found that the displacement of the shock response is decreased with the rise of $f_{d-in}$($f_{d-in}=\omega_{d-in}/2\pi$). Therefore, it is very critical to raising the in-phase frequency for enhancing the impact properties of micro-gyroscopes.

## 4. Architecture Design

Much research on the development and analysis of the silicon micro-gyroscope structure has been done in our previous work [24,25,26,27,28,29]. Therefore, this section mainly introduces the design and analysis of the coupling mechanism.

### 4.1. Design of the Coupling Mechanism

**Figure 5:**
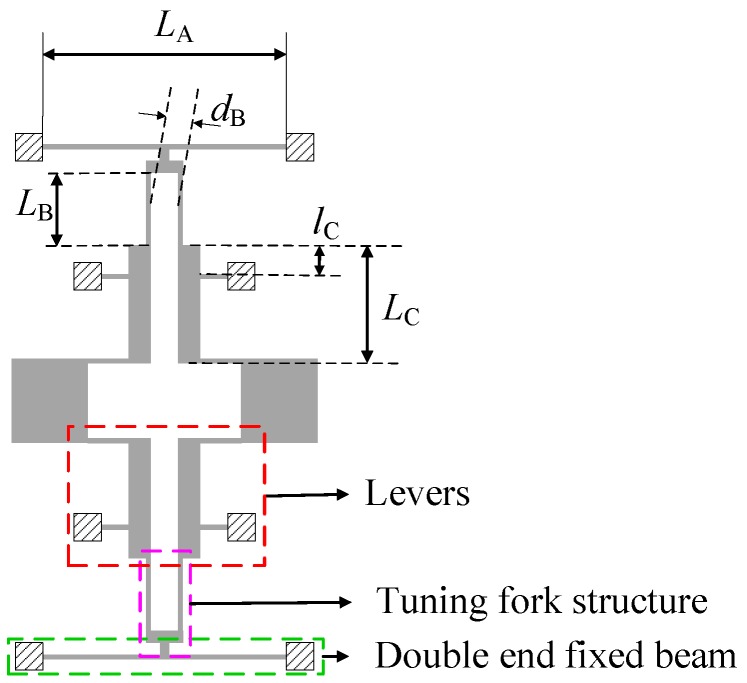
The proposed coupling mechanism.

According to the above analysis, it is necessary to design a coupling mechanism, for which the in-phase stiffness is greater than the anti-phase stiffness. Then, the in-phase mode frequency of the gyroscope would be higher than the anti-phase mode frequency, which could reduce the shock response without loss of mechanical sensitivity. A lever coupling mechanism is proposed based on the improved coupling method, as is shown in Figure 5. The architecture is completely symmetrical. The coupling structure is formed with three parts:

(1) levers, which are used to transform the displacement of the proof masses.
(2) a tuning fork structure, which is important to determine the values of the in-phase coupling stiffness $k_{in}$ and the anti-phase coupling stiffness $k_{an}$. In the in-phase mode, the two beams of the tuning fork resonator will be subjected to the in-phase forces along the x direction and will produce a torsional force around the z direction; while in the anti-phase mode, the two beams of the tuning fork resonator will be subjected to the anti-phase forces along the x direction and will produce an axial force in the y direction.
(3) a double-ended beam, which absorbs the force produced by the tuning fork structure. The different forces in the in- and anti-phase mode will cause the torsional deformation and the bending deformation, respectively.

**Figure 6:**
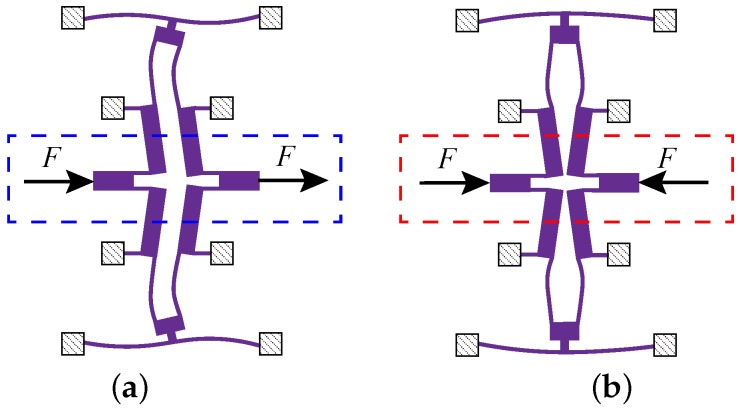
The two different mode shapes of the anchored diamond coupling beam in the in-phase mode (**a**) and anti-phase mode (**b**).

The coupling mechanism presents two different mode shapes in the in-phase mode and the anti-phase mode. For in-phase motion, the coupling mechanism is forced in opposite directions by the proof masses, and the double-ended beam presents bending deformation, as demonstrated in Figure 6a. For anti-phase motion, the coupling mechanism is forced in the same direction by the proof masses, and the double-ended beam presents torsional deformation, as depicted in Figure 6b. The parameters of the coupling structure will determine the in-phase mode and the anti-mode stiffness, which will be analyzed in the following section.

### 4.2. Analysis of the Relations between the Stiffness and the Structure Parameters

Generally, in order to improve the consistency of processing, the same width is applied to all slender beams. In this design, the width of the slender beam is 10 μm. This paper mainly introduces the effects of dimensions $L_{A}$, $L_{B}$, $d_{B}$, $L_{C}$ and $l_{C}$ on the in-phase and the anti-phase coupling stiffness. $L_{A}$ presents the length of the double-ended beam; $L_{B}$ presents the length of the tuning fork beam; $d_{B}$ presents the gap between the two tuning fork beams; $L_{C}$ presents the length of the lever; $l_{C}$ presents the length of the lever’s resisting arm. The initial values of $L_{A}$, $L_{B}$, $d_{B}$, $l_{C}$ and $L_{C}$ are shown in Table 2.

**Figure 7:**
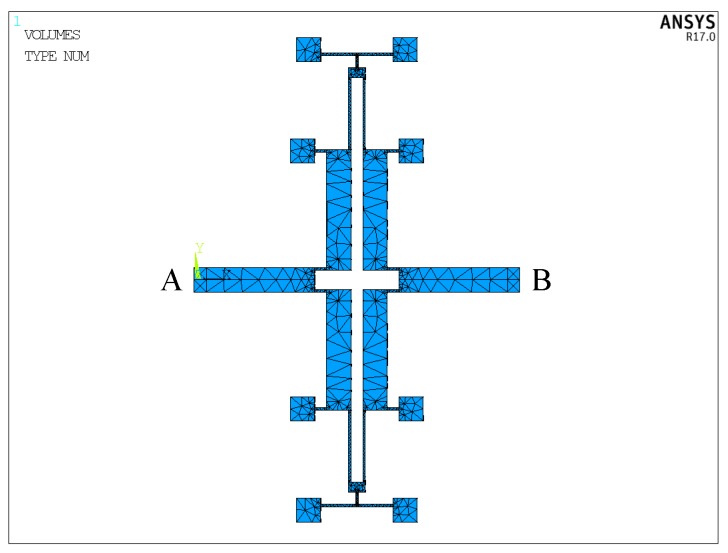
The finite element model of the lever coupling mechanism.

**Figure 8:**
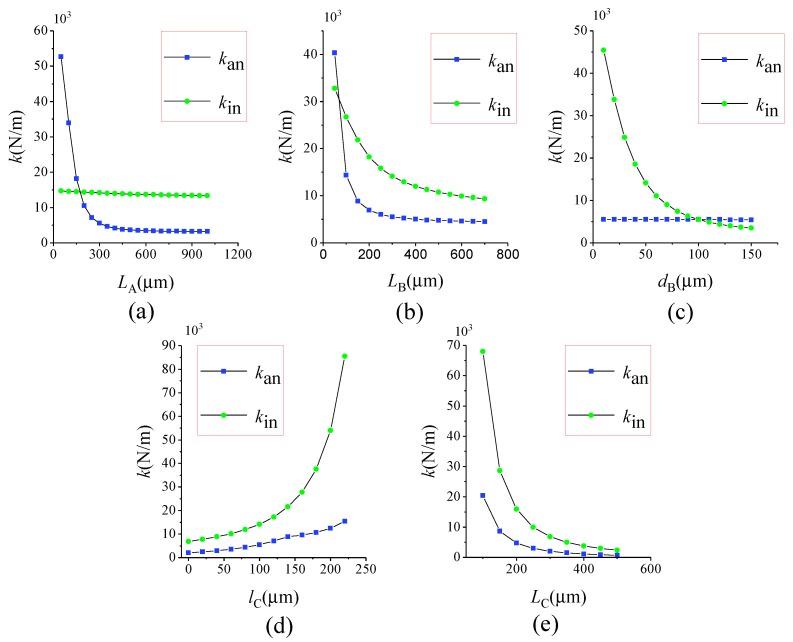
The relationships between the in-phase coupling stiffness, as well as the anti-phase coupling stiffness and: $L_{A}$ (**a**); $L_{B}$ (**b**); $d_{B}$ (**c**); $l_{C}$ (**d**); $L_{C}$ (**e**).

The stiffness can be calculated by formula k=F/Δx. Figure 7 shows the finite element model of the lever coupling mechanism. The thickness of the structure is 60 μm, and the mesh element is SOLID95. The element, which can tolerate irregular shapes without as much loss of accuracy, is defined by 20 nodes having three degrees of freedom per node: translations in the nodal x, y and z directions. The element may have any spatial orientation. SOLID95 has plasticity, creep, stress stiffening, large deflection and large strain capabilities. Various printout options are also available [30]. The silicon material properties are listed in Table 3 [31,32]. By applying a constant force on Surface-A and -B along the driving direction as shown in Figure 6, the displacement can be obtained by ANSYS static analysis. Then, the in-phase and anti-phase coupling stiffness are obtained. Figure 8 illustrates the relationship between the in-phase coupling stiffness, as well as the anti-phase coupling stiffness and $L_{A}$, $L_{B}$, $d_{B}$, $l_{C}$ and $L_{C}$, respectively.

**Figure 9:**
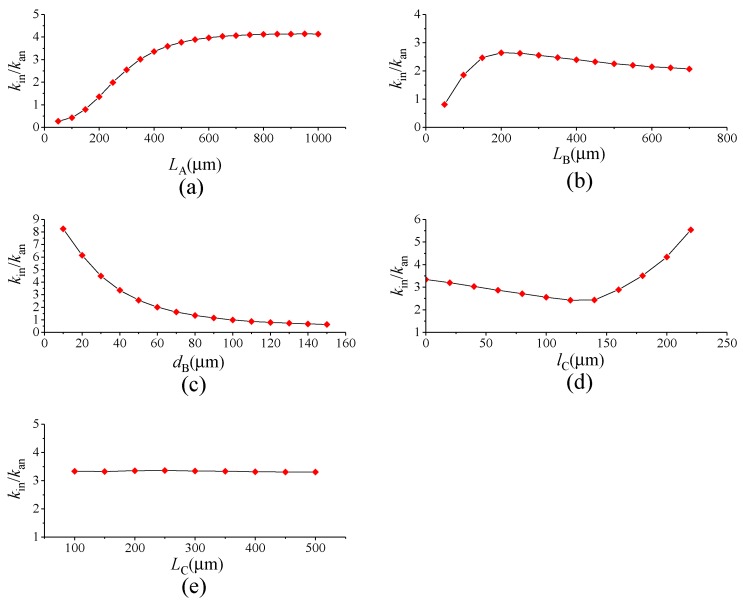
The relationship between the ratio of the in-phase coupling stiffness to the anti-phase coupling stiffness and: $L_{A}$ (**a**); $L_{B}$ (**b**); $d_{B}$ (**c**); $l_{C}$ (**d**); $L_{C}$ (**e**).

The ratio of in-phase coupling stiffness to the anti-phase coupling stiffness is shown in Figure 9. Figure 8a indicates that $L_{A}$ chiefly influences the anti-phase coupling stiffness, while $d_{B}$ principally decides the in-phase coupling stiffness, as described in Figure 8c. It can be seen from Figure 9b,d that the ratio shows a rapid increase at first and then decreases gradually as $L_{B}$ increases, yet it presents an increasing tendency after decreasing at the beginning as $l_{C}$ increases. Therefore, the maximum ratio can be obtained by optimizing $L_{B}$ and $l_{C}$. Figure 9e reveals that $L_{C}$ has an equivalent effect on both in-phase and anti-phase coupling stiffness. Therefore, the appropriate level of the in-phase and the anti-phase coupling stiffness can be gained by optimizing $L_{C}$.

In summary, the effect of $L_{A}$, $L_{B}$, $d_{B}$, $l_{C}$, $L_{C}$ on the in-phase coupling stiffness, as well as the anti-phase coupling stiffness is different. The ideal stiffness that we need can be obtained by adjusting their values according to the analyses above.

### 4.3. Architecture Design

**Figure 10:**
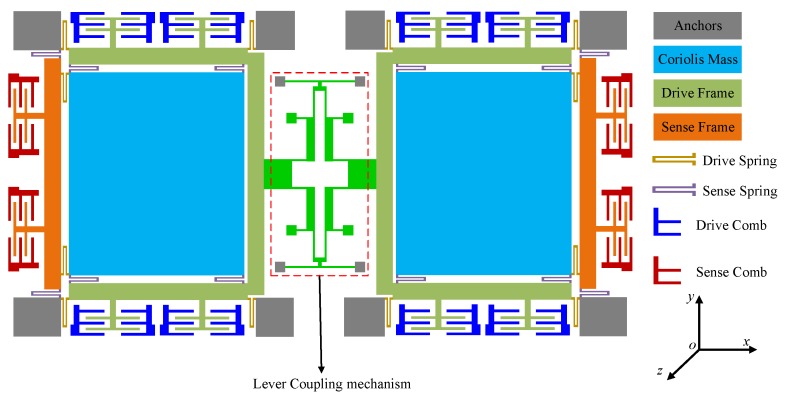
The silicon micro-gyroscope structure with the designed lever coupling mechanism.

Figure 10 shows the silicon micro-gyroscope structure with the designed lever coupling mechanism, which is based on the structure proposed in our previous work. Each tine includes a Coriolis proof mass and two frames, supported by symmetrical springs. The electrodes are variable-area capacitances to guarantee the linearity of the capacitance change with the displacement in the motion direction parallel to the plates.

## 5. FEM Simulations of the Whole Structure

**Figure 11:**
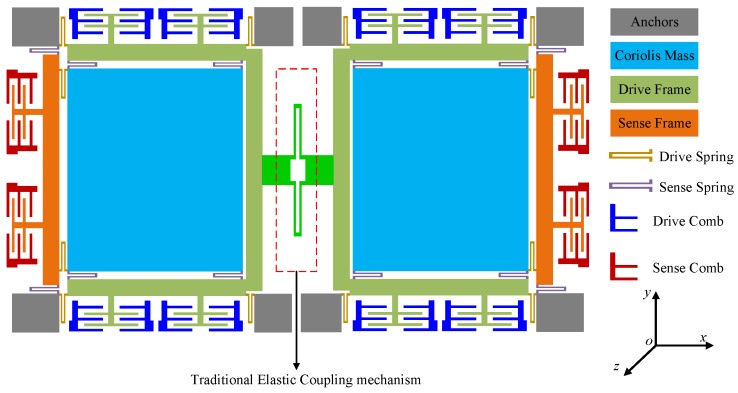
The dual-mass structure coupled via elastic springs.

A dual-mass structure with traditional coupling beams is given for contrast verification, as depicted in Figure 11. The architecture is the same as the silicon micro-gyroscope shown in Figure 10, except for the coupling part, where the direct coupling via elastic springs is adopted. This structure is defined as the Type-A structure, and the above structure is defined as the Type-B structure for convenience.

### 5.1. Modal Analysis

**Figure 12:**
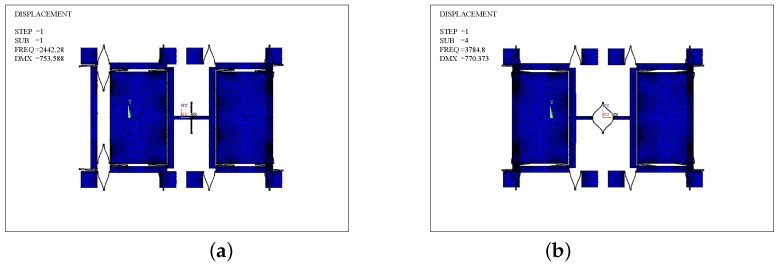
Modal analysis of the Type-A structure: (**a**) the in-phase mode; (**b**) the anti-phase mode.

The mode analyses of Type-A in the in- and anti-phase modes in the driving direction are carried out and shown in Figure 12a,b, and the mesh element is SOLID 95. The in- and anti-phase frequencies ($f_{d-in}$,$f_{d}$) are 2442.28 Hz and 3784.8 Hz, respectively. Similarly, the mode analysis of Type-B is made.

**Figure 13:**
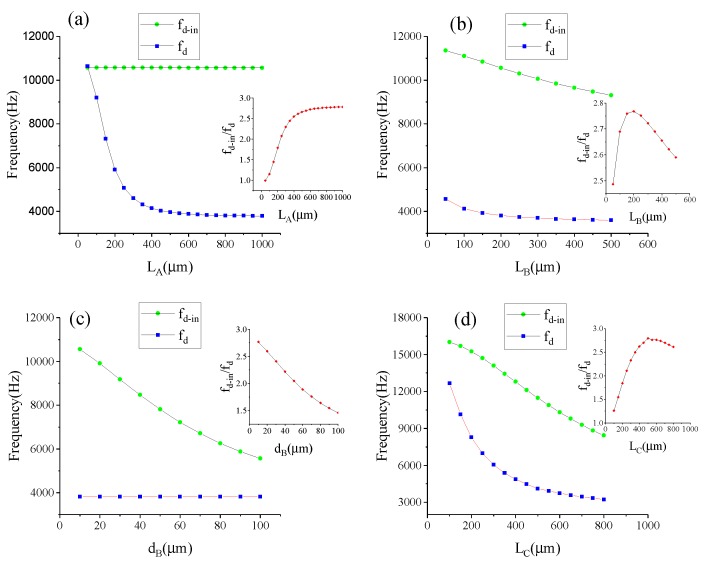
The relationships between $f_{d-in}$, $f_{d}$ and $f_{d-in}/f_{d}$ of Type-B and: $L_{A}$ (**a**); $L_{B}$ (**b**); $d_{B}$ (**c**); $L_{C}$ (**d**).

According to the analysis in Section 4.2, the $l_{C}$ value is zero to get a higher ratio, as well as to get a smaller anti-phase mode stiffness. Figure 13 shows the relationships between $f_{d-in}$, $f_{d}$ and $f_{d-in}/f_{d}$ of Type-B and $L_{A}$, $L_{B}$, $d_{B}$, $l_{C}$. It can be see that $L_{A}$ chiefly influences the value of $f_{d}$, while $d_{B}$ principally decides the value of $f_{d-in}$. $L_{B}$ and $L_{C}$ can influence both the $f_{d}$ and $f_{d-in}$ values. As depicted in the inset figures, the maximum ratio can be obtained by optimizing $L_{B}$ and $L_{C}$. According to the expressions $f_{d-in}=\frac{1}{2\pi }\sqrt{\frac{k_{d}+k_{in}}{m}}$ and $f_{d}=\frac{1}{2\pi }\sqrt{\frac{k_{d}+k_{an}}{m}}$, Figure 13 can confirm the trend in Figure 8 and Figure 9.

By adjusting and optimizing the dimensions of the coupling mechanism, its anti-phase frequency is approximately equal to that of Type-A. The final parameters of the lever coupling mechanism are given in Table 4.

**Figure 14:**
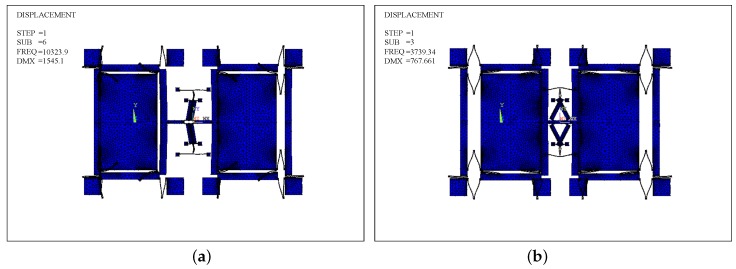
Modal analysis of the Type-B structure: (**a**) the in-phase mode; (**b**) the anti-phase mode.

Figure 14a,b presents the in-phase and anti-phase modes of Type-B. $f_{d-in}$ and $f_{d}$ are 10,323.9 Hz, 3739.34 Hz, respectively. It demonstrates that the Type-B architecture can optimize the modal order. Specifically, the in-phase mode frequency is nearly 176% higher than the anti-phase one. Because $f_{d}$ is almost the same in both Type-A and Type-B structures, as well as $\delta_{f}$ is uniform, the mechanical sensitivity of the Type-B has no loss compared with the Type-A.

### 5.2. Thermal Analysis Simulation

The mechanical and physical properties of the silicon material are greatly affected by the temperature. The change of temperature will lead to the structural deformation and the variation of the elastic modulus. In addition, due to the difference in the thermal expansion coefficient between the silicon structure layer and the glass substrate layer, the mismatch thermal stress will be produced as the temperature changes. These will cause the resonant frequency drift.

**Figure 15:**
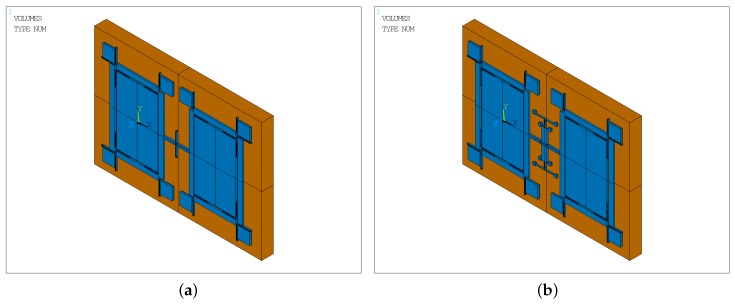
Simulation models of the two types of gyroscopes: (**a**) the Type-A structure; (**b**) the Type-B structure.

A thermal analysis simulation is performed for the two structures using the ANSYS software. Figure 15 shows the simulation models of the two types. In the figure, the blue part is a 60 μm-deep silicon layer, and the orange part is a 500 μm-deep glass layer. The mesh element is SOLID 90, which has 20 nodes with a single degree of freedom, temperature, at each node. The 20-node element has compatible temperature shapes, is well suited to model curved boundaries and is applicable to a 3D, steady-state or transient thermal analysis [30]. The change in stiffness due to thermal stress affects the natural frequency of the gyroscope.

Table 5 depicts the frequencies of the two gyroscopes at different temperatures. Table 5 shows that the working frequency shift of the Type-A structure is 10.71 Hz, whereas the frequency shift of the Type-B structure is 15.45 Hz. Obviously, although the proposed architecture is more complicated than the compared one (Type-A structure), the temperature performance is not deteriorated.

### 5.3. Transient Dynamic Analysis

There are three methods for the transient dynamics analysis: full method, reduced method and modal superposition method. The full method uses the full system matrix to calculate the transient response, which is the strongest of the three methods, allowing the inclusion of various non-linear properties (plasticity, large deformation, large strain) [33]. This paper uses the full method in the transient dynamics analysis.

The damping effect is crucial to determine the dynamic transient response. In this section, the full method analysis uses the Rayleigh damping model, which is one of the most widely-used damping models. Alpha damping and Beta damping are used to define Rayleigh damping constants α and β. The damping matrix [C] is calculated by using these constants to multiply the mass matrix [M] and stiffness matrix [K]: [C]=α[M]+β[K]. The values of α and β are not generally known directly, but can be calculated from modal damping ratios ($\xi_{i}$). $\xi_{i}$ is the ratio of actual damping to critical damping for a particular mode of vibration, i. If $\omega_{i}$ is the natural circular frequency of mode i, α and β satisfy the relation: $\xi_{i}=\alpha /2\omega_{i}+\beta \omega_{i}/2$. To specify both α and β for a given damping ratio ξ, it is commonly assumed that the sum of the α and β terms is nearly constant over a range of frequencies. Therefore, given ξ and a frequency range $\omega_{1}$ to $\omega_{2}$, two simultaneous equations can be solved for α and β [34]:

(11) $$\alpha =2\xi \frac{\omega_{1}\omega_{2}}{\omega_{1}+\omega_{2}}$$

(12) $$\beta =\frac{2\xi }{\omega_{1}+\omega_{2}}$$

According to our previous experimental results, the quality factor of the MEMS gyroscopes which we developed is about 3000–8000. Then, the modal damping ratio can be calculated by ξ=1/2Q, where, Q is the quality factor. In this paper, the quality factor uses 5000. The α and β values can be calculated by (11) and (12) based on the modal analysis results. The values of Type-A and Type-B structures are shown in Table 6.

#### 5.3.1. Impact Performance Comparison under the Same Shock Load

For verifying the theory analysis and the effectiveness of the proposed structure, transient dynamic analysis is implemented by using the ANSYS software. For the impact response we studied, there may occur negative volumes of the mesh element due to large deformation, which will lead the ANSYS calculation process to be terminated. In order to avoid the above situation, it is important to adjust and optimize the mesh size and accuracy.

**Figure 16:**
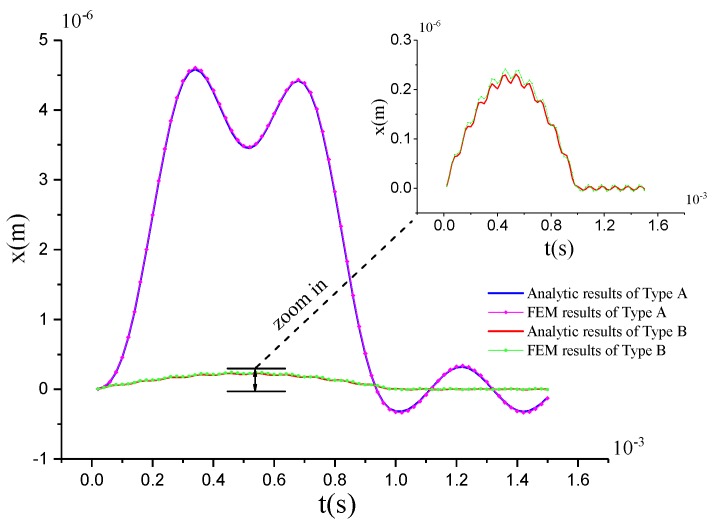
Analytic and FEM results of Type-A and Type-B.

In the simulation, shock load with peak acceleration $A_{p}=100$ g and duration time T=0.001 s is applied to both samples in the drive direction. Both the analytic results and the FEM results of two types are shown in Figure 16. Comparison of the peak of the shock response is provided in Table 7.

Of course, the analytic results and the FEM results coincide well with each other. The small difference between the analytic results and the FEM results is mainly caused by the neglecting of the non-linear effect in the analytic results and the equivalent treatments taken in model simplification and the unsymmetrical meshing in simulation modeling. Therefore, for the MEMS gyroscope with a good linearity, the mode superposition method can be used for the simplified analysis. However, to get the more accurate results, it is better to use the full method. Table 7 show that the shock response of Type-B is far lower than that of Type-A, which proves that the lever coupling mechanism is particularly effective at reducing the shock response.

**Figure 17:**
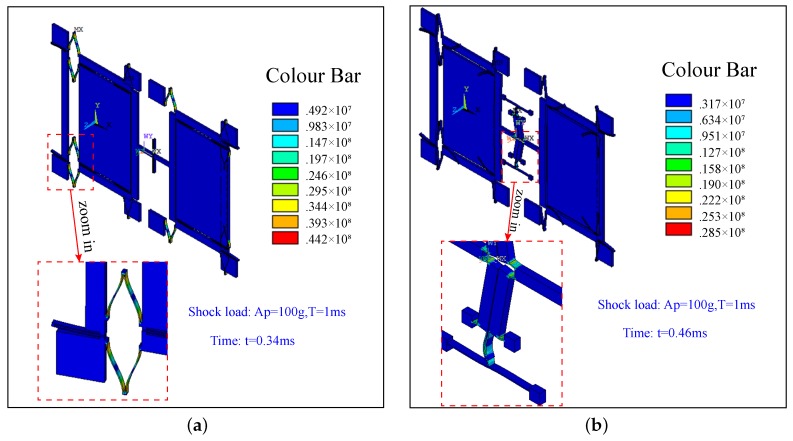
The stress distributions of Type-A (**a**) and Type-B (**b**) at the maximum impact displacement.

Figure 17 shows the stress distributions of Type-A (a) and Type-B (b) at the maximum impact displacement, respectively. It is obvious that the stress in Type-B is far lower than that in Type-A, which shows that the lever coupling mechanism is notably effective at reducing the stress caused by shock loads. Therefore, the Type-B structure with the designed coupling mechanism can truly reduce the impact response displacement and impact load stress.

#### 5.3.2. Shock Resistance of the Gyroscope along the *X*-Axis

**Figure 18:**
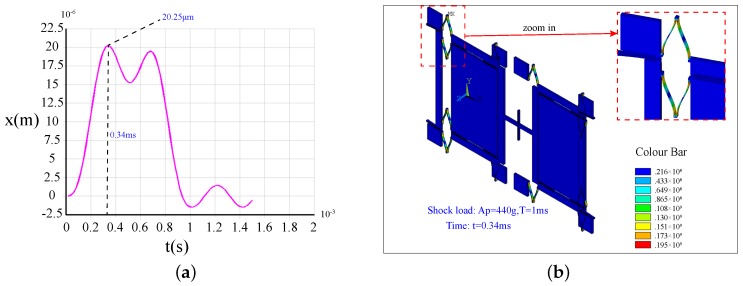
The shock response (**a**) and the stress distributions (**b**) at the maximum impact displacement of Type-A under a 440 g shock load.

Figure 18a illustrates the shock response of Type-A under a 440 g, 1-ms shock load. We can see that its peak value is 20.25μm, while the minimum clearance along the driving direction is 20μm. Therefore, the shock load should be less than this level to protect the structure against collisions. The stress distribution of Type-A at 0.28 ms (the moment of the maximum impact displacement) is shown in Figure 18b. It is obvious that the stress is far below the fracture strength of silicon materials (790 MPa) [29]. Therefore, for Type-A, the shock response has already reached the structure gap allowed, while the maximum structural stress value has not attained the ultimate stress. Therefore, the impact resistance ability is mainly determined by the allowed maximum shock displacement. It illustrates that the failure of this structure is mainly caused by the collision.

**Figure 19:**
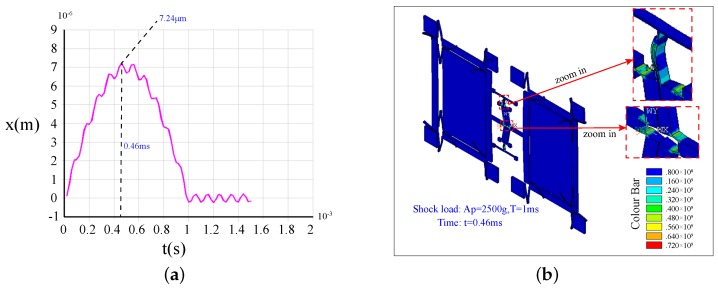
The shock response (**a**) and the stress distributions (**b**) at the maximum impact displacement of Type-B under a 2500 g shock load.

Figure 19a illustrates the shock response of Type-B under a 2500 g, 1-ms shock load. We can see that its peak value is 7.24μm, which is far less than 20μm. Figure 19b gives the stress distribution of Type-B at 0.46 ms (the moment of the maximum impact displacement). The biggest stress is about 720 MPa, which is lower than the allowable stress of silicon materials. The results show that the Type-B structure can withstand a 2500 g shock load. For Type-B, the fracture, which is caused by the stress concentration, is the main failure mode.

In summary, the shock resistance ability along the driving direction of Type-A is less than 400 g, while that of Type-B can reach 2500 g. The simulation results demonstrate that the lever coupling mechanism can significantly improve the shock resistance of the dual-mass silicon micro-gyroscope with drive mode coupled.

## 6. Discussion

Micro-gyroscope is an important part of MEMS inertial sensors. Shock resistance is one of the primary issues in the design of silicon micro-gyroscopes for many applications. For dual-mass bulk silicon micro-gyroscopes with drive mode coupled, the shock response along the driving direction can be enhanced by improving the in-phase mode frequency. However, generally, the gyroscope’s in-phase mode frequency is lower than and close to the anti-phase mode frequency, which is the operational mode frequency. Therefore, the improving of in-phase mode frequency will also raise the anti-phase mode frequency, which will influence the mechanical sensitivity. The coupling mechanism design method, which significantly improves the in-phase frequency, as well as keeps the anti-phase frequency almost constant, has its peculiar advantage in improving the shock resistance without sacrificing the mechanical sensitivity. In this paper, a new lever coupling mechanism is presented. The detailed comparisons of the pros and cons between this mechanism and other methods are listed in Table 8.

The critical dimensions of the lever coupling mechanism are discussed, and their effects on the in-phase stiffness and the anti-phase stiffness are discussed in detail. A micro-gyroscope equipped with the proposed lever coupling mechanism, which is based on our previous gyroscope structure, is designed. Modal analysis results show that the gyroscope’s in-phase mode frequency is 176% larger than the anti-phase one, while the anti-phase frequency is almost constant compared with the gyroscope with a traditional coupling mechanism. This verifies that the designed lever coupling mechanism has a significant effect on changing the modal order and improving the frequency separation. Compared with the contrast structure, the mechanical sensitivity has no loss. Due to the mechanical and physical properties of the silicon material being greatly affected by the temperature, the temperature performance is also analyzed. Although the proposed architecture is more complicated than the compared one, the results showed that the temperature performance was not deteriorated. The shock response of the designed dual-mass silicon micro-gyroscope and the contrast structure under the same specified shock load (100 g, 1 ms) along the driving direction is calculated by using both the analytic solution and the FEM method. The correctness of the analytic solution is verified well. Both the analytic solution and FEM method conclusions prove that the silicon micro-gyroscope structure with the lever coupling mechanism can notably reduce the shock response by more than one order of magnitude and cut down the stress produced in the shock process by about 35.5% compared with the traditional elastic coupled one. In addition, the shock resistance of both structures is investigated. The results show that the shock resistance of the improved gyroscope can reach 2500 g along the driving direction, while that of the traditional one is less than 440 g. Consequently, the lever coupling mechanism is able to change the modal order and improve the frequency separation by structurally offering a higher stiffness difference ratio and tremendously enhances the shock resistance in the driving direction without sacrificing sensitivity.

Because this design is based on the gyroscope we previously developed, which is only coupled in the drive motion, the coupling mechanism can only improve the shock resistance along the driving direction. Its shock resistance along the sensing direction should be enhanced by using stoppers or the optimization of beams, which will be studied deeply in our future work. The lever coupling mechanism is universal for a dual-mass silicon micro-gyroscope with drive mode coupled. Furthermore, for a micro-gyroscope with both drive and sense mode coupled, the research on the sense mode coupling mechanism is necessary and significant to improve the shock resistance. 

## Figures and Tables

**Table 1:** The parameters used for the simulation.

Parameters	$m_{d}$ (kg)	$A_{p}$(g)	*T*(s)	ω(rad/s)
Values	$8.798\times 10^{-7}$	100	0.001	1000π

**Table 2:** The parameters of the coupling mechanism used for the simulation.

Parameters	$L_{A}$(μm)	$L_{B}$(μm)	$d_{B}$(μm)	$L_{C}$(μm)	$l_{C}$(μm)
Values	300	300	50	300	100

**Table 3:** Model parameters for FEM simulations.

Parameters	Young’s Modulus (Pa)	Poisson’s Ratio	Density (kg/m^3^)
Values	$1.7\times 10^{11}$	0.28	2330

**Table 4:** The parameters of the lever coupling mechanism.

Parameters	$L_{A}$(μm)	$L_{B}$(μm)	$d_{B}$(μm)	$L_{C}$(μm)	$l_{C}$(μm)
Values	800	400	10	580	0

**Table 5:** The frequencies of the two structures at different temperatures ($f_{d}$).

Frequency (Hz)	Type-A	Type-B
−40 °C	3769.12	3754.48
−20 °C	3767.10	3762.62
0 °C	3765.02	3767.73
20 °C	3762.87	3770.36
40 °C	3760.67	3770.95
60 °C	3758.41	3769.93

**Table 6:** The α and β values of Type-A and Type-B structures.

Value	α	β
Type-A	0.297	$3.21\times 10^{-8}$
Type-B	0.549	$1.42\times 10^{-8}$

**Table 7:** The peak of the shock response in the analytic and FEM results.

Peak Value (μm)	Analytic Results	FEM Results
Type-A	4.604	4.584
Type-B	0.2294	0.2414

**Table 8:** The detailed comparisons between the proposed structure and other methods.

Methods	Advantages	Disadvantages
Improving the robustness of the weak structures	Simple to design, easy to implement	Slight effect
The use of stoppers	Simple to design, easy to implement	Easy to cause fracture, debris, and performance shifts
The traditional method to raise the in-phase mode frequency	Simple to design	Reduce the mechanical sensitivity
The ring or diamond coupling structures	Improve the shock resistance without sacrificing the mechanical sensitivity	Complicated structure, stringent machining precision requirements
The proposed coupling structure	Improve the shock resistance without sacrificing the mechanical sensitivity, easy to process	Complicated structure

## Appendix A

By mode superposition Method [35], (9) can be decoupled and simplified as:

(A1) $$\ddot{q}\left(t\right)+2\zeta_{1}\omega_{d-in}\dot{q}\left(t\right)+\omega_{d-in}^{2}q\left(t\right)=-a\left(t\right)\sqrt{2m_{d}},$$

(A2) $$\ddot{q}\left(t\right)+2\zeta_{2}\omega_{d}\dot{q}\left(t\right)+\omega_{d}^{2}q\left(t\right)=0,$$

It can be found that the shock response of the structure is only decided by the in-phase mode response. (A1) can be expressed as:

(A3) $$\ddot{q}\left(t\right)+2\zeta_{1}\omega_{d-in}\dot{q}\left(t\right)+\omega_{d-in}^{2}q\left(t\right)=-A_{p}\sqrt{2m_{d}}sin\omega t,0\leq t\leq T,$$

(A4) $$\ddot{q}\left(t\right)+2\zeta_{1}\omega_{d-in}\dot{q}\left(t\right)+\omega_{d-in}^{2}q\left(t\right)=0,t\geq T.$$

Laplace transform is applied to solve (A3); the result of the transformation is given by:

(A5) $$\begin{array}{cc} Q\left(s\right)=A_{p}\sqrt{2m_{d}}\omega \{ & \frac{C_{1}}{s-(-\zeta_{1}\omega_{d-in}-j\omega_{d-in}\sqrt{1-{\zeta_{1}}^{2}})}\\ & +\frac{C_{2}}{s-(-\zeta_{1}\omega_{d-in}+j\omega_{d-in}\sqrt{1-{\zeta_{1}}^{2}})}+\frac{C_{3}}{s-j\omega }+\frac{C_{4}}{s+j\omega }\}, \end{array}$$

where,

(A6) $$C_{1}=\frac{1}{4\zeta_{1}{\omega_{d-in}}^{3}\left(1-{\zeta_{1}}^{2}\right)-j2\omega_{d-in}\sqrt{1-{\zeta_{1}}^{2}}\left(2{\zeta_{1}}^{2}{\omega_{d-in}}^{2}-{\omega_{d-in}}^{2}+\omega^{2}\right)},$$

(A7) $$C_{2}=\frac{1}{4\zeta_{1}{\omega_{d-in}}^{3}\left(1-{\zeta_{1}}^{2}\right)+j2\omega_{d-in}\sqrt{1-{\zeta_{1}}^{2}}\left(2{\zeta_{1}}^{2}{\omega_{d-in}}^{2}-{\omega_{d-in}}^{2}+\omega^{2}\right)},$$

(A8) $$C_{3}=\frac{1}{4\zeta_{1}\omega_{d-in}\omega^{2}+j2\omega \left({\omega_{d-in}}^{2}-\omega^{2}\right)},$$

(A9) $$C_{4}=\frac{1}{4\zeta_{1}\omega_{d-in}\omega^{2}-j2\omega \left({\omega_{d-in}}^{2}-\omega^{2}\right)}.$$

Then, the solution to (A3) is derived by the inverse Laplace transformation, which is given below:

(A10) $$q\left(t\right)=A_{p}\sqrt{2m_{d}}\omega \left(C_{1}e^{(-\zeta_{1}\omega_{d-in}-j\omega_{d-in}\sqrt{1-{\zeta_{1}}^{2}})t}+C_{2}e^{(-\zeta_{1}\omega_{d-in}+j\omega_{d-in}\sqrt{1-{\zeta_{1}}^{2}})t}+C_{3}e^{j\omega t}+C_{4}e^{-j\omega t}\right),$$

Similarly, the solution to (A4) can be obtained by the same method.

The modal vector matrix is expressed as [ϕ]. The shock response can be calculated by x(t)=[ϕ]q(t) and is given by:

(A11) $$\begin{array}{c} x\left(t\right)=\left\{\begin{array}{cc} \begin{array}{c} -\frac{A_{p}e^{-\zeta_{1}\omega_{d-in}t}}{\omega_{d-in}\sqrt{1-\zeta_{1}^{2}}}\frac{1}{\omega^{4}-2\omega^{2}\omega_{d-in}^{2}\left(1-2\zeta_{1}^{2}\right)+\omega_{d-in}^{4}}\{2\zeta_{1}\omega \omega_{d-in}^{2}\sqrt{1-\zeta_{1}^{2}}-\\ e^{\zeta_{1}\omega_{d-in}t}cos\left(\omega t\right)+\omega_{d-in}^{3}e^{\zeta_{1}\omega_{d-in}t}\left[{\sqrt{1-\zeta_{1}^{2}}}^{3}+\zeta_{1}^{2}\left(1-\zeta_{1}^{2}\right)-\sqrt{1-\zeta_{1}^{2}}\right]sin\left(\omega t\right)+\\ \left[\omega^{3}-\omega \omega_{d-in}^{2}\left(1-\zeta_{1}^{2}\right)+\omega \omega_{d-in}^{2}\zeta_{1}^{2}\right]sin\left(\omega_{d-in}\sqrt{1-\zeta_{1}^{2}}t\right)\} \end{array} & 0\leq t\leq T,\\ \begin{array}{c} -\frac{A_{p}e^{-\zeta_{1}\omega_{d-in}t}}{\omega_{d-in}\sqrt{1-\zeta_{1}^{2}}}\frac{1}{\omega^{4}-2\omega^{2}\omega_{d-in}^{2}\left(1-2\zeta_{1}^{2}\right)+\omega_{d-in}^{4}}\cdot \\ \{2\zeta_{1}\omega \omega_{d-in}^{2}\sqrt{1-\zeta_{1}^{2}}\left[cos\left(\omega_{d-in}\sqrt{1-\zeta_{1}^{2}}t\right)+e^{\zeta_{1}\omega_{d-in}t}cos\left(\omega_{d-in}\sqrt{1-\zeta_{1}^{2}}\left(t-T\right)\right)\right]+\\ \left[\omega^{3}-\omega \omega_{d-in}^{2}\left(1-\zeta_{1}^{2}\right)+\omega \omega_{d-in}^{2}\zeta_{1}^{2}\right][sin\left[\omega_{d-in}\sqrt{1-\zeta_{1}^{2}}t\right]+\\ e^{\zeta_{1}\omega_{d-in}t}sin\left(\omega_{d-in}\sqrt{1-\zeta_{1}^{2}}\left(t-T\right)\right)]\} \end{array} & t\geq T. \end{array}\right. \end{array}$$
